# Dibromidobis(1-ethyl-2,6-dimethyl­pyridinium-4-olate-κ*O*)zinc(II)

**DOI:** 10.1107/S1600536810052190

**Published:** 2010-12-18

**Authors:** M. Thenmozhi, A. Philominal, S. Dhanuskodi, M. N. Ponnuswamy

**Affiliations:** a Centre of Advanced Study in Crystallography and Biophysics, University of Madras, Guindy Campus, Chennai 600 025, India; b Department of Physics, Bharathidasan University, Tiruchirappalli 620 024, India

## Abstract

In the bioactive title compound, [ZnBr_2_(C_9_H_13_NO)_2_], the Zn^II^ atom is coordinated in a distorted tetra­hedral arrangement by two Br^−^ anions and the O atoms of two zwitterionic organic ligands. The pyridinium rings are almost planar [maximum deviations = 0.004 (4) and 0.003 (4) Å]. The ethyl groups are approximately perpendicular to the corresponding pyridinium ring planes [N—C—C—C = 88.8 (4)° in each ligand]. The packing of the mol­ecules is controlled by π–π inter­actions, with centroid–centroid distances of 3.625 (3) and 3.711 (2) Å, forming chains approximately parallel to (102). The crystal studied was non-merohedrally twinned (twin relationship between the domains 1 0 0, 0 1 0, −0.4672 −0.1864 −1 and batch scale factor of 7.39%).

## Related literature

For general background to pyridinium compounds and their applications, see: Darensbourg *et al.* (2003 ▶); Dhanuskodi *et al.* (2006 ▶); Glavcheva *et al.* (2004 ▶); Lakshmanaperumal *et al.* (2002 ▶, 2004 ▶); Usman *et al.* (2000 ▶, 2001 ▶); Mootz & Wusson (1981 ▶). For their biological activity, see: Akkurt *et al.* (2005 ▶). For related structures, see: Thenmozhi *et al.* (2010 ▶); Mootz & Wusson (1981 ▶); Sundar *et al.* (2004 ▶). For the preparation of the ligand, see: Garratt (1963 ▶).
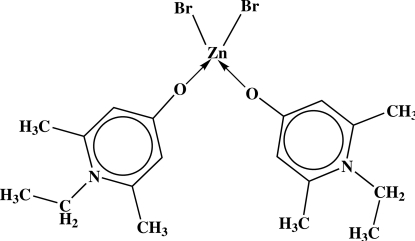


## Experimental

### 

#### Crystal data

[ZnBr_2_(C_9_H_13_NO)_2_]
*M* *_r_* = 527.60
Triclinic, 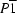

*a* = 8.462 (1) Å
*b* = 8.518 (1) Å
*c* = 14.418 (3) Å
α = 93.131 (6)°
β = 97.871 (7)°
γ = 90.210 (8)°
*V* = 1027.9 (2) Å^3^
*Z* = 2
Mo *K*α radiation
μ = 5.10 mm^−1^
*T* = 293 K
0.12 × 0.11 × 0.11 mm

#### Data collection

Bruker Kappa APEXII area-detector diffractometer
Absorption correction: multi-scan (*SADABS*; Sheldrick, 2001 ▶) *T*_min_ = 0.580, *T*_max_ = 0.604
16553 measured reflections
16553 independent reflections
13614 reflections with *I* > 2σ(*I*)

#### Refinement

*R*[*F* ^2^ > 2σ(*F* ^2^)] = 0.059
*wR*(*F* ^2^) = 0.189
*S* = 1.06
16553 reflections
233 parameters
H-atom parameters constrained
Δρ_max_ = 1.20 e Å^−3^
Δρ_min_ = −0.95 e Å^−3^

### 

Data collection: *APEX2* (Bruker, 2004 ▶); cell refinement: *SAINT* (Bruker, 2004 ▶); data reduction: *SAINT*; program(s) used to solve structure: *SHELXS97* (Sheldrick, 2008 ▶); program(s) used to refine structure: *SHELXL97* (Sheldrick, 2008 ▶); molecular graphics: *ORTEP-3* (Farrugia, 1997 ▶); software used to prepare material for publication: *SHELXL97* and *PLATON* (Spek, 2009 ▶).

## Supplementary Material

Crystal structure: contains datablocks global, I. DOI: 10.1107/S1600536810052190/sj5050sup1.cif

Structure factors: contains datablocks I. DOI: 10.1107/S1600536810052190/sj5050Isup2.hkl

Additional supplementary materials:  crystallographic information; 3D view; checkCIF report

## Figures and Tables

**Table 1:** Selected bond lengths (Å)

O1—Zn1	1.957 (3)
O2—Zn1	1.976 (3)
Zn1—Br2	2.3501 (8)
Zn1—Br1	2.3635 (8)

**Fig. 1.**
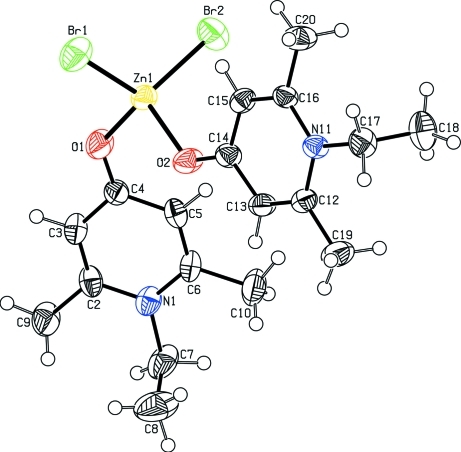
A perspective view of the molecule with displacement ellipsoids drawn at the 50% probability level.

**Fig. 2.**
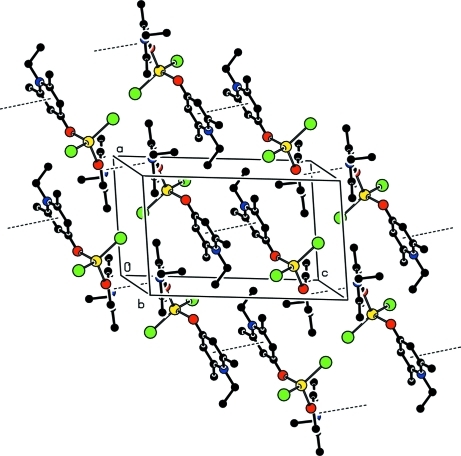
Crystal packing of the title compound with all H atoms omitted for clarity.

## supplementary crystallographic information

### Comment

Pyridinium derivatives are found to possess nonlinear optical properties (Lakshmanaperumal *et al.*, 2002, 2004; Usman *et al.*, 2000, 2001). When pyridinium cations are combined with metal halide anions, the refractive indices of the crystals could be tuned due to exchangeability of metal and halogen species within the anions (Glavcheva *et al.*, 2004). Halide anions have been reported to improve the physicochemical stability of 1-ethyl-2, 6-dimethyl-4-(1H)- pyridinones (Dhanuskodi *et al.*, 2006). Reactions of  zinc halides with pyridines lead to a variety of complexes involving zinc centers and were shown to be catalytically active (Darensbourg *et al.*, 2003). Pyridinium derivatives also exhibit antibacterial and antifungal activities (Akkurt *et al.*, 2005). As a part of our interest in the bioactivity of pyridinium complexes, we report here the crystal structure of the title compound, Fig. 1.

The bromidozinc complex is similar to the related chlorido complex, bis(1-ethyl-2,6-dimethylpyridinium-4-oxide-κO)dichloridozinc(II) (Thenmozhi *et al.*, 2010). The pyridinium rings are planar and oriented at an angle of 34.4 (2)° to one another. The Zn^II^ atom is coordinated in a distorted tetrahedral arrangement by two halide ions and two zwitterionic pyridinium oxide ligands. The pyridinium rings assume a substantial degree of quinoidal character, which is reflected in the variation of bond lengths (Sundar *et al.*, 2004). The ethyl groups attached at N1 and N11 are approximately perpendicular to pyridinium ring, which can be observed from the torsion angles [C8-C7-N1-C2 = -88.5 (6)°; C18-C17-N11-C12 = 87.6 (5)°]. The methyl substituents at C2, C6, C12 and C16 are nearly coplanar with the corresponding pyridinium rings, which is evident from the torsion angles [C9-C2-N1-C6 = 178.0 (4)°; C10-C6-N1-C2 = -178.4 (4)°; C19-C12-N11-C16 = 179.9 (4)°; C20-C16-N11-C12 = -178.2 (4)°]. Due to protonation of N1 and N11 atoms of the pyridinium rings, the C2-N1-C6 and C12-N11-C16 angles [Table 1] are widened in comparison with the literature value (Mootz & Wusson, 1981). The sum of the bond angles around the protonated nitrogen atoms N1[359.8°] and N11[360.0°] of both the pyridinium rings is in accordance with *sp**^2^* character.

The packing of the molecules is reinforced by π-π intermolecular interactions. [Cg1···Cg1(2-x, 1-y, -z) = 3.625 (3)Å; where Cg1 is the centroid of the (N1-C6) ring] and [Cg2···Cg2(1-x, 1-y, 1-z) = 3.711 (2)Å; where Cg2 is the centroid of the (N11-C16) ring]. The π–π interactions generate infinite continuous chains approximately parallel to (102), Fig.2.

### Experimental

The complex was prepared by the reaction of ZnBr_2 _with 1-ethyl-2, 6-dimethyl -4(1H) pyridinone trihydrate (EDMP.3H_2_O) in a 1:2 molar ratio in aqueous medium. The starting material EDMP.3H_2_O had been prepared by the reported synthetic method (Garratt *et al.*, 1963). The salts were further purified by the repeated recrystallization in triple distilled water. The solubility test of the salts were carried out by mass gravimetric method in the temperature range 30°-55°C and water is the suitable solvent for the growth of good quality crystals. Single crystals of (EDMP)_2_ZnBr_2_ were harvested after a typical growth period of 15 days from the saturated aqueous solution at 30°C by the slow evaporation of the solvent.

### Refinement

H atoms were positioned geometrically (C-H = 0.93-0.97Å) and allowed to ride on their parent atoms, with U_iso_(H) = 1.5U_eq_(C) for methyl H and 1.2U_eq_(C) for other H atoms. The crystal was non-merohedrally twinned with the twin relationship between the domains 1 0 0, 0 1 0, -0.4672 -0.1864 -1 and the batch scale factor is of 7.39%.

### Crystal data

**Table:**

[ZnBr_2_(C_9_H_13_NO)_2_]	*Z* = 2
*M**_r_* = 527.60	*F*(000) = 528
Triclinic, *P*1	*D*_x_ = 1.705 Mg m^−^^3^
Hall symbol: -P 1	Mo *K*α radiation, λ = 0.71073 Å
*a* = 8.462 (1) Å	Cell parameters from 16553 reflections
*b* = 8.518 (1) Å	θ = 1.4–25.0°
*c* = 14.418 (3) Å	µ = 5.10 mm^−^^1^
α = 93.131 (6)°	*T* = 293 K
β = 97.871 (7)°	Block, colourless
γ = 90.210 (8)°	0.12 × 0.11 × 0.11 mm
*V* = 1027.9 (2) Å^3^	

### Data collection

**Table:**

Bruker Kappa APEXII area-detector diffractometer	16553 independent reflections
Radiation source: fine-focus sealed tube	13614 reflections with *I* > 2σ(*I*)
graphite	*R*_int_ = 0.000
ω and φ scans	θ_max_ = 25.0°, θ_min_ = 1.4°
Absorption correction: multi-scan (*SADABS*; Sheldrick, 2001)	*h* = −10→10
*T*_min_ = 0.580, *T*_max_ = 0.604	*k* = −10→10
16553 measured reflections	*l* = −17→17

### Refinement

**Table:**

Refinement on *F*^2^	Primary atom site location: structure-invariant direct methods
Least-squares matrix: full	Secondary atom site location: difference Fourier map
*R*[*F*^2^ > 2σ(*F*^2^)] = 0.059	Hydrogen site location: inferred from neighbouring sites
*wR*(*F*^2^) = 0.189	H-atom parameters constrained
*S* = 1.06	*w* = 1/[σ^2^(*F*_o_^2^) + (0.0512*P*)^2^ + 13.5867*P*] where *P* = (*F*_o_^2^ + 2*F*_c_^2^)/3
16553 reflections	(Δ/σ)_max_ < 0.001
233 parameters	Δρ_max_ = 1.20 e Å^−^^3^
0 restraints	Δρ_min_ = −0.95 e Å^−^^3^

### Special details

**Table:**

Geometry. All esds (except the esd in the dihedral angle between two l.s. planes) are estimated using the full covariance matrix. The cell esds are taken into account individually in the estimation of esds in distances, angles and torsion angles; correlations between esds in cell parameters are only used when they are defined by crystal symmetry. An approximate (isotropic) treatment of cell esds is used for estimating esds involving l.s. planes.
Refinement. Refinement of F^2^ against ALL reflections. The weighted R-factor wR and goodness of fit S are based on F^2^, conventional R-factors R are based on F, with F set to zero for negative F^2^. The threshold expression of F^2^ > 2sigma(F^2^) is used only for calculating R-factors(gt) etc. and is not relevant to the choice of reflections for refinement. R-factors based on F^2^ are statistically about twice as large as those based on F, and R- factors based on ALL data will be even larger.

### Fractional atomic coordinates and isotropic or equivalent isotropic displacement parameters (Å^2^)

**Table:**

	*x*	*y*	*z*	*U*_iso_*/*U*_eq_	
C2	1.2061 (5)	0.5686 (6)	0.1164 (3)	0.0379 (11)	
C3	1.1571 (5)	0.4291 (5)	0.1424 (3)	0.0369 (11)	
H3	1.2321	0.3503	0.1536	0.044*	
C4	0.9992 (5)	0.3972 (5)	0.1532 (3)	0.0350 (10)	
C5	0.8909 (5)	0.5207 (5)	0.1318 (3)	0.0372 (11)	
H5	0.7830	0.5052	0.1351	0.045*	
C6	0.9418 (5)	0.6608 (6)	0.1066 (3)	0.0383 (11)	
C7	1.1564 (7)	0.8459 (6)	0.0842 (4)	0.0565 (15)	
H7A	1.0751	0.8990	0.0437	0.068*	
H7B	1.2507	0.8371	0.0530	0.068*	
C8	1.1965 (8)	0.9428 (7)	0.1766 (5)	0.080 (2)	
H8A	1.1046	0.9476	0.2089	0.120*	
H8B	1.2276	1.0473	0.1642	0.120*	
H8C	1.2828	0.8946	0.2149	0.120*	
C9	1.3764 (6)	0.5978 (6)	0.1057 (4)	0.0535 (14)	
H9A	1.4400	0.5118	0.1293	0.080*	
H9B	1.4132	0.6935	0.1404	0.080*	
H9C	1.3858	0.6069	0.0406	0.080*	
C10	0.8236 (6)	0.7901 (7)	0.0860 (4)	0.0620 (16)	
H10A	0.7186	0.7529	0.0922	0.093*	
H10B	0.8256	0.8212	0.0231	0.093*	
H10C	0.8513	0.8786	0.1293	0.093*	
C12	0.4359 (5)	0.6653 (5)	0.3969 (3)	0.0337 (10)	
C13	0.5639 (5)	0.6198 (5)	0.3569 (3)	0.0360 (10)	
H13	0.6383	0.6954	0.3469	0.043*	
C14	0.5894 (5)	0.4615 (5)	0.3295 (3)	0.0339 (10)	
C15	0.4688 (5)	0.3538 (5)	0.3449 (3)	0.0331 (10)	
H15	0.4788	0.2481	0.3273	0.040*	
C16	0.3392 (5)	0.4008 (5)	0.3848 (3)	0.0317 (9)	
C17	0.1813 (5)	0.6083 (6)	0.4556 (3)	0.0429 (11)	
H17A	0.2108	0.6970	0.4997	0.051*	
H17B	0.1447	0.5237	0.4900	0.051*	
C18	0.0477 (6)	0.6553 (7)	0.3824 (4)	0.0587 (15)	
H18A	0.0911	0.7110	0.3353	0.088*	
H18B	−0.0248	0.7219	0.4114	0.088*	
H18C	−0.0081	0.5628	0.3538	0.088*	
C19	0.4154 (6)	0.8323 (6)	0.4274 (4)	0.0537 (14)	
H19A	0.4116	0.8405	0.4938	0.080*	
H19B	0.3178	0.8707	0.3950	0.080*	
H19C	0.5036	0.8937	0.4133	0.080*	
C20	0.2141 (6)	0.2841 (6)	0.3993 (4)	0.0513 (13)	
H20A	0.2376	0.1835	0.3714	0.077*	
H20B	0.1118	0.3182	0.3706	0.077*	
H20C	0.2124	0.2755	0.4653	0.077*	
O1	0.9580 (4)	0.2656 (4)	0.1836 (3)	0.0475 (9)	
O2	0.7136 (4)	0.4211 (4)	0.2935 (2)	0.0429 (8)	
Zn1	0.75971 (6)	0.21641 (6)	0.23222 (4)	0.03594 (15)	
Br1	0.81438 (7)	0.03353 (6)	0.34965 (4)	0.05785 (18)	
Br2	0.55242 (6)	0.15373 (7)	0.10973 (4)	0.05343 (17)	
N1	1.0984 (4)	0.6872 (4)	0.1000 (2)	0.0371 (9)	
N11	0.3231 (4)	0.5561 (4)	0.4125 (2)	0.0315 (8)	

### Atomic displacement parameters (Å^2^)

**Table:**

	*U*^11^	*U*^22^	*U*^33^	*U*^12^	*U*^13^	*U*^23^
C2	0.029 (2)	0.051 (3)	0.035 (2)	0.007 (2)	0.0074 (19)	0.008 (2)
C3	0.034 (2)	0.044 (3)	0.035 (2)	0.010 (2)	0.0085 (19)	0.011 (2)
C4	0.030 (2)	0.035 (3)	0.042 (3)	−0.0005 (19)	0.0070 (19)	0.006 (2)
C5	0.026 (2)	0.047 (3)	0.038 (3)	0.005 (2)	0.0040 (19)	0.002 (2)
C6	0.034 (2)	0.042 (3)	0.039 (3)	0.014 (2)	0.003 (2)	0.008 (2)
C7	0.060 (3)	0.044 (3)	0.073 (4)	0.011 (3)	0.026 (3)	0.029 (3)
C8	0.087 (5)	0.050 (4)	0.112 (6)	−0.017 (3)	0.045 (4)	0.004 (4)
C9	0.039 (3)	0.055 (3)	0.072 (4)	0.007 (2)	0.023 (3)	0.021 (3)
C10	0.044 (3)	0.055 (4)	0.091 (5)	0.020 (3)	0.013 (3)	0.026 (3)
C12	0.039 (3)	0.028 (2)	0.034 (2)	0.0002 (19)	0.0043 (19)	0.0002 (19)
C13	0.036 (2)	0.029 (2)	0.044 (3)	−0.0046 (19)	0.008 (2)	0.000 (2)
C14	0.033 (2)	0.033 (2)	0.035 (2)	−0.0012 (19)	0.0053 (19)	−0.0052 (19)
C15	0.041 (3)	0.028 (2)	0.029 (2)	0.0029 (19)	0.0046 (19)	−0.0016 (18)
C16	0.032 (2)	0.031 (2)	0.030 (2)	−0.0044 (18)	−0.0020 (18)	−0.0008 (18)
C17	0.038 (3)	0.048 (3)	0.044 (3)	0.004 (2)	0.013 (2)	−0.004 (2)
C18	0.043 (3)	0.077 (4)	0.058 (3)	0.014 (3)	0.010 (3)	0.006 (3)
C19	0.055 (3)	0.031 (3)	0.075 (4)	−0.002 (2)	0.017 (3)	−0.009 (3)
C20	0.049 (3)	0.038 (3)	0.071 (4)	−0.013 (2)	0.023 (3)	−0.001 (3)
O1	0.0378 (18)	0.040 (2)	0.070 (2)	0.0061 (15)	0.0231 (17)	0.0082 (17)
O2	0.0421 (19)	0.0312 (18)	0.058 (2)	0.0000 (14)	0.0203 (16)	−0.0084 (15)
Zn1	0.0333 (3)	0.0302 (3)	0.0456 (3)	0.0014 (2)	0.0118 (2)	−0.0023 (2)
Br1	0.0676 (4)	0.0476 (3)	0.0622 (4)	0.0091 (3)	0.0173 (3)	0.0164 (3)
Br2	0.0441 (3)	0.0648 (4)	0.0494 (3)	−0.0022 (2)	0.0027 (2)	−0.0056 (3)
N1	0.041 (2)	0.038 (2)	0.034 (2)	0.0061 (17)	0.0099 (17)	0.0120 (17)
N11	0.0326 (19)	0.031 (2)	0.0302 (19)	−0.0020 (15)	0.0055 (15)	−0.0048 (15)

### Geometric parameters (Å, °)

**Table:**

C2—C3	1.347 (6)	C12—C19	1.484 (6)
C2—N1	1.371 (5)	C13—C14	1.410 (6)
C2—C9	1.492 (6)	C13—H13	0.9300
C3—C4	1.395 (6)	C14—O2	1.275 (5)
C3—H3	0.9300	C14—C15	1.418 (6)
C4—O1	1.289 (5)	C15—C16	1.358 (6)
C4—C5	1.417 (6)	C15—H15	0.9300
C5—C6	1.352 (6)	C16—N11	1.374 (5)
C5—H5	0.9300	C16—C20	1.493 (6)
C6—N1	1.360 (6)	C17—N11	1.483 (5)
C6—C10	1.504 (6)	C17—C18	1.508 (7)
C7—N1	1.476 (6)	C17—H17A	0.9700
C7—C8	1.525 (9)	C17—H17B	0.9700
C7—H7A	0.9700	C18—H18A	0.9600
C7—H7B	0.9700	C18—H18B	0.9600
C8—H8A	0.9600	C18—H18C	0.9600
C8—H8B	0.9600	C19—H19A	0.9600
C8—H8C	0.9600	C19—H19B	0.9600
C9—H9A	0.9600	C19—H19C	0.9600
C9—H9B	0.9600	C20—H20A	0.9600
C9—H9C	0.9600	C20—H20B	0.9600
C10—H10A	0.9600	C20—H20C	0.9600
C10—H10B	0.9600	O1—Zn1	1.957 (3)
C10—H10C	0.9600	O2—Zn1	1.976 (3)
C12—C13	1.344 (6)	Zn1—Br2	2.3501 (8)
C12—N11	1.379 (5)	Zn1—Br1	2.3635 (8)
			
C3—C2—N1	119.8 (4)	O2—C14—C15	123.5 (4)
C3—C2—C9	121.2 (4)	C13—C14—C15	115.4 (4)
N1—C2—C9	119.0 (4)	C16—C15—C14	121.9 (4)
C2—C3—C4	123.0 (4)	C16—C15—H15	119.1
C2—C3—H3	118.5	C14—C15—H15	119.1
C4—C3—H3	118.5	C15—C16—N11	120.0 (4)
O1—C4—C3	121.4 (4)	C15—C16—C20	120.3 (4)
O1—C4—C5	123.3 (4)	N11—C16—C20	119.7 (4)
C3—C4—C5	115.3 (4)	N11—C17—C18	111.4 (4)
C6—C5—C4	121.1 (4)	N11—C17—H17A	109.3
C6—C5—H5	119.4	C18—C17—H17A	109.3
C4—C5—H5	119.4	N11—C17—H17B	109.3
C5—C6—N1	121.1 (4)	C18—C17—H17B	109.3
C5—C6—C10	119.5 (4)	H17A—C17—H17B	108.0
N1—C6—C10	119.4 (4)	C17—C18—H18A	109.5
N1—C7—C8	111.1 (4)	C17—C18—H18B	109.5
N1—C7—H7A	109.4	H18A—C18—H18B	109.5
C8—C7—H7A	109.4	C17—C18—H18C	109.5
N1—C7—H7B	109.4	H18A—C18—H18C	109.5
C8—C7—H7B	109.4	H18B—C18—H18C	109.5
H7A—C7—H7B	108.0	C12—C19—H19A	109.5
C7—C8—H8A	109.5	C12—C19—H19B	109.5
C7—C8—H8B	109.5	H19A—C19—H19B	109.5
H8A—C8—H8B	109.5	C12—C19—H19C	109.5
C7—C8—H8C	109.5	H19A—C19—H19C	109.5
H8A—C8—H8C	109.5	H19B—C19—H19C	109.5
H8B—C8—H8C	109.5	C16—C20—H20A	109.5
C2—C9—H9A	109.5	C16—C20—H20B	109.5
C2—C9—H9B	109.5	H20A—C20—H20B	109.5
H9A—C9—H9B	109.5	C16—C20—H20C	109.5
C2—C9—H9C	109.5	H20A—C20—H20C	109.5
H9A—C9—H9C	109.5	H20B—C20—H20C	109.5
H9B—C9—H9C	109.5	C4—O1—Zn1	127.6 (3)
C6—C10—H10A	109.5	C14—O2—Zn1	128.4 (3)
C6—C10—H10B	109.5	O1—Zn1—O2	101.12 (13)
H10A—C10—H10B	109.5	O1—Zn1—Br2	111.24 (11)
C6—C10—H10C	109.5	O2—Zn1—Br2	108.65 (10)
H10A—C10—H10C	109.5	O1—Zn1—Br1	108.88 (10)
H10B—C10—H10C	109.5	O2—Zn1—Br1	108.20 (10)
C13—C12—N11	120.3 (4)	Br2—Zn1—Br1	117.46 (3)
C13—C12—C19	120.9 (4)	C6—N1—C2	119.7 (4)
N11—C12—C19	118.8 (4)	C6—N1—C7	120.7 (4)
C12—C13—C14	122.4 (4)	C2—N1—C7	119.4 (4)
C12—C13—H13	118.8	C16—N11—C12	120.0 (3)
C14—C13—H13	118.8	C16—N11—C17	120.3 (4)
O2—C14—C13	121.1 (4)	C12—N11—C17	119.7 (4)
			
N1—C2—C3—C4	0.5 (7)	C14—O2—Zn1—O1	172.7 (4)
C9—C2—C3—C4	179.9 (5)	C14—O2—Zn1—Br2	55.6 (4)
C2—C3—C4—O1	−175.8 (5)	C14—O2—Zn1—Br1	−72.9 (4)
C2—C3—C4—C5	2.1 (7)	C5—C6—N1—C2	2.1 (7)
O1—C4—C5—C6	175.2 (5)	C10—C6—N1—C2	−178.3 (5)
C3—C4—C5—C6	−2.7 (7)	C5—C6—N1—C7	−172.2 (5)
C4—C5—C6—N1	0.7 (7)	C10—C6—N1—C7	7.4 (7)
C4—C5—C6—C10	−178.9 (5)	C3—C2—N1—C6	−2.6 (7)
N11—C12—C13—C14	−0.2 (7)	C9—C2—N1—C6	177.9 (4)
C19—C12—C13—C14	178.2 (5)	C3—C2—N1—C7	171.7 (5)
C12—C13—C14—O2	−178.5 (4)	C9—C2—N1—C7	−7.7 (7)
C12—C13—C14—C15	1.5 (7)	C8—C7—N1—C6	85.7 (6)
O2—C14—C15—C16	179.0 (4)	C8—C7—N1—C2	−88.6 (5)
C13—C14—C15—C16	−1.0 (6)	C15—C16—N11—C12	2.2 (6)
C14—C15—C16—N11	−0.8 (6)	C20—C16—N11—C12	−178.2 (4)
C14—C15—C16—C20	179.6 (4)	C15—C16—N11—C17	179.7 (4)
C3—C4—O1—Zn1	164.0 (3)	C20—C16—N11—C17	−0.8 (6)
C5—C4—O1—Zn1	−13.8 (7)	C13—C12—N11—C16	−1.8 (6)
C13—C14—O2—Zn1	−169.5 (3)	C19—C12—N11—C16	179.9 (4)
C15—C14—O2—Zn1	10.5 (6)	C13—C12—N11—C17	−179.2 (4)
C4—O1—Zn1—O2	−33.4 (4)	C19—C12—N11—C17	2.5 (6)
C4—O1—Zn1—Br2	81.8 (4)	C18—C17—N11—C16	−89.8 (5)
C4—O1—Zn1—Br1	−147.2 (4)	C18—C17—N11—C12	87.6 (5)

### Hydrogen-bond geometry (Å, °)

**Table:**

*D*—H···*A*	*D*—H	H···*A*	*D*···*A*	*D*—H···*A*
C5—H5···O2	0.93	2.57	3.093 (5)	116.
